# Parental Genetic Variants, MTHFR 677C>T and MTRR 66A>G, Associated Differently with Fetal Congenital Heart Defect

**DOI:** 10.1155/2017/3043476

**Published:** 2017-07-03

**Authors:** Qian-nan Guo, Hong-dan Wang, Li-zhen Tie, Tao Li, Hai Xiao, Jian-gang Long, Shi-xiu Liao

**Affiliations:** ^1^The Medical Genetic Center, Henan Provincial People's Hospital, People's Hospital of Zhengzhou University, Zhengzhou 450000, China; ^2^Center for Mitochondrial Biology and Medicine, The Key Laboratory of Biomedical Information Engineering of Ministry of Education, School of Life Science and Technology, Xi'an Jiaotong University, Xi'an 710049, China; ^3^Henan Chengxin Institute of Forensic Clinical Judicial Authentication, Zhengzhou 450003, China; ^4^Department of Cardiology, Henan Provincial People's Hospital, People's Hospital of Zhengzhou University, Zhengzhou 450000, China

## Abstract

**Background:**

Congenital heart defect (CHD) is one of the most common birth defects in the world. The methylenetetrahydrofolate reductase (MTHFR) and methionine synthase reductase (MTRR) genes are two of the most important candidate genes for fetal CHD. However, the correlations between the two genes and fetal CHD were inconsistent in various reports. Therefore, this study is aimed to evaluate the parental effects of the two genes on fetal CHD via three genetic polymorphisms, MTHFR 677C>T (rs1801133), MTHFR 1298 A>C (rs1801131), and MTRR 66A>G (rs1801394).

**Methods:**

Parents with pregnancy history of fetal CHD were divided into two subgroups: ventricular septal defect (VSD) (21) and non-VSD groups (78). VSD, non-VSD, and 114 control parents (controls) were analyzed in this study. Genotyping of these genetic polymorphisms was done by sequencing.

**Results:**

The MTHFR 677C>T polymorphism of either mothers or fathers was independently associated with fetal non-VSD (*P* < 0.05) but not VSD, while the MTRR 66A>G polymorphism was independently associated with fetal VSD (*P* < 0.05) but not non-VSD. No significance was found for MTHFR 1298A>C polymorphism.

**Conclusion:**

In either maternal or paternal group, the MTHFR 677C>T polymorphism was independently related to fetal non-VSD, while the MTRR 66A>G polymorphism was independently related to fetal VSD.

## 1. Introduction

Around the world, periconceptional folic acid intake in females is thought to reduce the risk of CHD [1] in the newborn. Therefore, interest in the genetic susceptibility to CHD has led to a growing attention to the study of polymorphisms of genes involved in folate metabolism, especially the two key genes—MTHFR and MTRR.

In recent years, many studies reported that two genetic polymorphisms of the two genes, MTHFR 677C>T and MTRR 66A>G, could cause elevated blood homocysteine (Hcy) level in human [2–6]. In several human studies [7–9], the elevated maternal blood Hcy level or amniotic fluid Hcy level was correlated with CHD in embryo. Moreover, high dosage Hcy injection treatment in avian embryo led to embryonic VSD [10]. Therefore, MTHFR and MTRR deficiency seemed to have adverse effects on fetal CHD development and probably via their effects in affecting Hcy level in both pregnant women and embryos.

Several studies reported that the two MTHFR 677C>T and MTHFR 1298 A>C genetic polymorphisms of both pregnant women and fetuses were related to fetal CHD [11–13]; however, some other studies showed contradictory results. For example, in a small Italian population study [14] and a large population metastudy [7], these two studies reported that both maternal and fetal MTHFR 677C>T and MTHFR 1298 A>C polymorphisms were not related to fetal CHD. Moreover, some studies reported that only maternal MTHFR 677C>T was associated with fetal CHD but not MTHFR 1298A>C [15, 16].

In several transgenic mice model experiments, MTRR gene with a hypomorphic mutation led to both embryo heart defect and hyperhomocysteinemia (hHcy) [17–19]. The hHcy resulting from the hypomorphic mutation in mice was similar to MTRR 66A>G mutation induced by elevated blood Hcy level in human. Therefore, MTRR 66A>G polymorphism in human also seemed to have a relationship with human heart defect. However, in human studies, some reported MTRR 66A>G was related to CHD development [20–22], while the others were contradictory [23, 24].

In a transgenic mice model experiment [19], it revealed that the mothers with a MTRR deficiency had an association with fetal VSD phenotype in mice. This result suggested that maternal MTRR deficiency might strongly be related to fetal VSD rather than other CHD types. In order to investigate whether this hypothetical relationship between maternal MTRR and fetal VSD exists in human and whether this relationship also occurs between father and fetus, and furthermore to analyze whether the parental MTHFR 677C>T and MTHFR 1298A>C polymorphisms' effects on fetal CHD in human were also VSD specific, parents with specific fetal VSD-diagnosed pregnancy history (VSD parents) and other types of CHD pregnancy history (non-VSD parents) were both studied in this experiment.

## 2. Materials and Methods

### 2.1. Subjects

Statement of responsibility: the authors had full access to and take full responsibility for the integrity of the data. All authors have read and agree to the manuscript as written.

Parental subjects with Chinese Han nationality were recruited from Middle China, Henan province, the second most populous province in China.

Study subjects (CHD parents and control parents): from January, 2014, to February, 2016, study subjects were recruited from the Department of Gynaecology and Obstetrics, Cardiac Center, and the Prenatal Diagnosis Center (also called the Medical Genetic Center) in the Henan Provincial People's Hospital. Written informed consent form was obtained from all participants.

CHD parents were divided into two subgroups: VSD parents and non-VSD parents. VSD parents were 21 couples with pregnancy history of CHD that occurred in fetuses or children which was specifically diagnosed as VSD. Non-VSD parents were 78 couples with pregnancy history of other types of CHD rather than VSD that occurred in fetuses or children (CHD classification; see Table 1). The CHD parents were at ages between 24 and 34 years (see Table 2) and had at least once pregnancy of CHD child, with no obvious fat or emaciation, no drug abuse history, no hypertension, no heart defect, no medical treatment during pregnancy, no pregnancy-induced hypertension, no diabetes, no smoking, no alcohol abuse, no family history of any diseases.

Control parents: the control parents were at ages between 20 and 34 years (mean ± SD: mothers 27.781 ± 2.205, fathers 29.789 ± 2.585, Table 2) and had, at least once, normal pregnancy of healthy babies and had no abnormal pregnancy history or had not given birth to abnormal children and had no hypertension, no heart defect, no medical treatment during pregnancy, no diabetes, no pregnancy-induced hypertension, no obvious fat, no drug abuse history, no smoking, no alcohol abuse, and no family history of any diseases.

All the control and CHD subjects recruited in this study and their babies/fetus were all diagnosed in the Prenatal Diagnosis Center (also called the Medical Genetic Center) by karyotype analysis of peripheral blood or amniotic fluid. The exactly genetic causation of CHD is unclear and about ~50% Down syndrome patients [25] have CHD; therefore, the parents or their babies/fetus who have abnormal karyotypes were excluded from this study.

The controls and cases were all unrelated Chinese Han nationality who lived in the middle of China, Henan province, had no preconceptional intake of folic acid, only with folic acid taken at 400 ng/day~800 ng/day since they knew they were pregnant and it is about more one month later after gestation. The mothers within any group studied here were all under 35 years old. The father and mother had well matched ages and BMI between the two study groups and the father had a generally larger age and bigger BMI value than the mother within each group (Table 2).

Total 232 CHD parents and 259 control parents were randomly selected, but only 21 VSD, 78 non-VSD, and 114 control couples fit the above requirements and were studied in this experiment.

### 2.2. Sample Collection

DNA was extracted from peripheral blood samples drawn into 2 ml tubes containing EDTA and was stored at −20°C.

### 2.3. Genotype Analysis

All the three polymorphisms were analyzed by polymerase chain reaction (PCR) followed by Sanger Chain Terminal sequencing method. Genomic DNA was amplified by Eppendorf (Master cycler gradient, Germany).

#### 2.3.1. Determination of MTHFR 677C>T Polymorphism

Primers used were as follows: forward primer 5′-gaagcagggagctttgaggctg3′ and reverse primer 5′-cccatgtcggtgcatgccttc3′ (made by Shanghai Sangon Biotech company, China). The PCR products were subjected to direct sequencing by using the reverse primer.

#### 2.3.2. Determination of MTHFR 1298A>C Polymorphism

Primers used were as follows: forward primer 5′gggaggagctgaccagtgaag3′ and reverse primer 5′ggggtcaggccaggggcag3′ (made by Shanghai Sangon Biotech company, China). The PCR products were subjected to direct sequencing by using the forward primer.

#### 2.3.3. Determination of MTRR 66A>G Polymorphism

Primers used were described as in Gaughan DJ [6] (made by Shanghai Sangon Biotech company, China). The PCR products were subjected to direct sequencing by using the reverse primer.

### 2.4. Statistical Analysis

Statistical significance of the differences in the frequency of alleles and genotypes using the Chi-square test was applied using the Statistical Package for Social Sciences (SPSS) version 13.0 statistical software (SPSS, Chicago, USA).

Odds ratio (OR) and 95% confidence intervals (95% CI) were calculated when it was appropriate to assess the relative risk conferred by a particular allele and genotype. Hardy-Weinberg equilibrium was tested for goodness-of-fit Chi-square test to compare the observed genotype frequencies among the subjects with the expected genotype frequencies.

A *p* value less than 0.05 (*P* < 0.05) was considered to be statistically significant.

## 3. Results

The genotype distributions of the MTHFR 677C>T, MTHFR 1298 A>C, and MTRR 66A>G polymorphisms were all in Hardy-Weinberg equilibrium in both mothers and fathers within either control or the two CHD parental subgroups (*P* > 0.05).

### 3.1. Allele Distribution in Father and Mother (Table 3 and Table 4)

There were no significant differences between mothers and fathers within the same group in both control and CHD parents (*P* > 0.05) (Table 3).

The frequency of MTHFR 677T allele was significantly higher in the non-VSD parents than the controls (mother: non-VSD 63.5% versus control 47.4%, *P* < 0.05; fathers: non-VSD 59.6% versus control 46.9%, *P* < 0.05). Therefore, either the maternal or the paternal MTHFR 677T allele was the independent risk factor for fetal non-VSD (mother: OR = 1.930, 95% CI: 1.272–2.928; father: OR = 1.669, 95% CI: 1.105–2.521; *P* < 0.05) (Tables 3 and 4).

The frequency of MTRR 66G allele was significantly higher in the VSD parents than the control parents (mothers: VSD 40.5% versus control 21.9%, *P* < 0.05; fathers: VSD 38.1% versus control 21.1%, *P* < 0.05). Therefore, either the maternal or the paternal MTRR 66G allele was the independent risk factor for fetal VSD (mother: OR = 2.421, 95% CI: 1.213–4.833; father: OR = 2.308, 95% CI: 1.147–4.645; *P* < 0.05) (Tables 3 and 4).

The parental allele frequencies of MTHFR 1298 A>C polymorphism were not significantly different between control and the two CHD groups (Tables 3 and 4).

### 3.2. Genotype Distribution in Mother (Table 5) and Father (Table 6)

The frequency of MTHFR 677TT genotype was significantly higher in both non-VSD fathers and mothers (mothers: non-VSD 42.3% versus controls 26.3%, *P* < 0.05; fathers: non-VSD 34.6% versus controls 26.3%, *P* < 0.05). The frequency of CT genotype was significantly higher only in non-VSD fathers (fathers: non-VSD 50.0% versus controls 41.2%, *P* < 0.05). In mothers, TT genotype has 3.3 times the risk (OR = 3.300, 95% CI: 1.454–7.488, *P* < 0.05) of CC genotype for causing a non-VSD pregnancy. In fathers, TT genotype has 2.775 times the risk (OR = 2.775, 95% CI: 1.206–6.385, *P* < 0.05) of CC genotype for causing a non-VSD pregnancy, CT genotype has 2.559 times the risk (OR = 2.559, 95% CI: 1.176–5.566, *P* < 0.05) of CC genotype for causing a non-VSD pregnancy.

The frequency of MTRR 66GG genotype was significantly higher in VSD mothers (VSD 14.3% versus control 7.7%, *P* < 0.05) and GG genotype has 9.571 times the risk (OR = 9.571, 95% CI: 1.615–56.736, *P* < 0.05) of CC genotype for causing a VSD pregnancy.

The genotype frequencies of MTHFR 1298A>C polymorphism were unrelated to both fetal VSD and non-VSD in either mothers or fathers.

## 4. Discussion

In this study, all subjects recruited had no preconceptional folic acid intake and only had folic acid intake after more than 1 month pregnancy; this requirement would significantly help to avoid the folic acid supplement effect on reducing fetal CHD [1]. Therefore, the conclusion made from this experiment should be meaningful.

### 4.1. Parental MTHFR 677C>T Polymorphism Causes Fetal Non-VSD but Not VSD

Both MTHFR 677C>T [2] and 1298 A>C [26] polymorphisms have been expressed and confirmed to affect MTHFR enzyme activity. 677TT enzyme had a 70% activity reduction, 677CT enzyme had a 35% activity reduction, and 1298CC enzyme had a 40% activity reduction. However, only MTHFR 677C>T and not 1298A<C polymorphism was associated with hHcy [27]. In human, the MTRR 66A>G polymorphism was also related to hHcy [6, 28, 29], similar as observed in MTRR deficient transgenic mice [17–19].

Hcy can be harmful to cells because it evokes oxidative stress through the production of reactive oxidative stress through the production of reactive oxygen species, binds to nitric oxide, or leads to the accumulation of its precursor, S-adenosylhomocysteine, a protein inhibitor of biological transmethylation. A study [9] reported that the maternal hHcy was correlated with fetal CHD; therefore, maternal MTHFR 677C>T and MTRR 66A>G polymorphisms, which would induce high blood level of Hcy, should have effects on fetal CHD development. However, in this study, the independent maternal and paternal MTHFR 677C>T polymorphism but not the MTRR 66A>G polymorphism was associated with fetal non-VSD; this suggested that maternal and paternal hHcy might not be a major risk factor for fetal non-VSD. There might exist another important mechanism pathway via MTHFR deficiency causing consequence results rather than hHcy alone. This hypothesis could be supported by the finding observed in the chicken embryo study where Hcy injection into chicken embryo led to majorly (83%) subarterial VSD [30].

Therefore, the effect of MTHFR 677C>T polymorphism on the development of fetal non-VSD observed in this study was probably via impaction on the synthesis of purine, thymidylate, or DNA methylation pathway rather than Hcy alone implicated in the folate metabolic pathway.

Mice lacking the MTHFR gene [31] displayed delayed development, impaired growth, and increased morbidity and mortality in the early postnatal period. In women, the 677C>T mutation was a risk factor for fetal neural tube defects (NTD), fetal Down syndrome, and recurrent embryo loss. Therefore, female MTHFR 677C>T polymorphism might have an impact not only on fetal survival but also on quality of embryo probably via impact on female germ cell development. Moreover, severe MTHFR deficiency in male mice resulted in abnormal spermatogenesis and infertility [32]. In human males, the aberrant promoter hypermethylation of MTHFR gene [33, 34] and the MTHFR 677C>T polymorphism [34–36] were both believed to be strongly associated with male infertility; MTHFR 677C>T polymorphism was also believed to be associated with abnormal spermatogenesis [34], semen quality (motility and morphology) [32, 34], and embryo heart defect [20]; MTHFR might control the DNA integrity and the function of sperm in human males [37, 38]. Therefore, MTHFR 677C>T polymorphism might also affect germ cell development in males. Thus, the effect of MTHFR 677C>T polymorphism on fetal CHD might have its impact on both female and male germ cell development.

However, the MTHFR 677C>T polymorphism only resulted in fetal non-VSD but not VSD observed in the study was a new proposal among the current world studies and the reasons or mechanisms for this were unclear. This proposal could explain why the relationship between MTHFR 677C>T and fetal CHD was varied among different studies due to different proportion of non-VSD subjects included in different population studies. In this study, as the MTHFR 677C>T polymorphism effect on fetal non-VSD was maternal and paternal independent, thus, either the maternal or paternal MTHFR 677C>T polymorphism was the risk factor for fetal non-VSD.

### 4.2. Parental MTRR 66A>G Polymorphism Causes Fetal VSD but Not Non-VSD

Studies in chicken embryos showed 83% subarterial VSD after injection of 30 *μ*M Hcy into the neural tube lumen [30]. This Hcy concentration resembled mild hHcy in humans. In the transgenic mice model, maternal MTRR gene with a hypomorphic mutation led to hHcy [18] and almost VSD phenotype in the mice embryo as well [19]. Therefore, maternal MTRR deficiency seemed to be strongly associated with fetal VSD phenotype, and this was probably due to hHcy effect resulting from MTRR deficiency. In this study, either the maternal or paternal MTRR 66A>G polymorphism was related to fetal VSD but not fetal non-VSD; this observation was consistent with the hypothesis that the maternal MTRR 66A>G polymorphism was strongly associated with fetal VSD. However, no animal experiment has been done to support the observation found in this experiment where the paternal MTRR 66A>G polymorphism was also strongly associated with fetal VSD rather than non-VSD. The mechanism underlining this is unclear.

### 4.3. Conclusion

Overall, in this study, the MTHFR 677C>T polymorphism of either mother or father was independently associated with fetal non-VSD but not VSD; fetal VSD was linked with the independent maternal or paternal MTRR 66A>G polymorphism. These findings made from this study could help to explain why the relationship between the two polymorphisms (MTHFR 677C>T and MTRR 66A>G) and CHD were varied among different studies due to different proportion of VSD and non-VSD subjects included in those different population studies.

Furthermore, a metastudy with selection of a larger sample size on VSD and non-VSD parents if possible could be considered in the future to further confirm the conclusion made from this study.

## Figures and Tables

**Table 1:** Classification of congenital heart disease.

CHD types	PA	DORV	TGA	TOF	UVH	SA	IPDA	AVS	PVS	VSD
CHD groups	*Non-VSD parents (78)*									*VSD parents (21)*
Number	5	2	3	22	7	8	22	4	5	21
%	6.4	2.6	3.8	28.2	9.0	10.2	28.2	5.1	6.4	100

Note: PA: pulmonary atresia, DORV: double outlet of right ventricular, TGA: arteries transposition, TOF: tetralogy of Fallot, UVH; univentricular hearts, SA: single atrium, IPDA: isolated patent ductus arteriosus, AVS: aortic valve stenosis, PVS: pulmonary valve stenosis, and VSD: ventricular septal defect.

**Table 2:** BMI and age distribution.

		Father (kg/m^2^)	*P*_1_	Mother (kg/m^2^)	*P*_1_	*P*_2_
BMI	Control	23.308 ± 2.607		21.345 ± 2.210		1.110*E* − 06
	Non-VSD	23.165 ± 3.093	0.739	21.761 ± 2.143	0.193	9.114*E* − 05
	VSD	23.136 ± 2.245	0.967	21.172 ± 2.275	0.254	0.008
Age	Control	29.789 ± 2.585		27.781 ± 2.205		9.063*E* − 39
	Non-VSD	30.256 ± 2.525	0.216	28.077 ± 2.278	0.368	2.256*E* − 30
	VSD	30.714 ± 2.305	0.128	28.714 ± 1.953	0.072	1.082*E* − 07

Note: BMI value is represented as mean ± SD; *P*_1_ is the 2-tail unpaired *T*-test within mothers or fathers between control and CHD group; *P*_2_ is the 2-tail paired *T*-test between mothers and fathers within the same group.

**Table 3:** Distribution of MTHFR 677C>T, MTHFR 1298A>C, and MTRR 66A>G polymorphisms.

		Control (114)				Non-VSD (78)				VSD (21)			
		Mother	Father	*χ* ^2^_1_	*P*_1_	Mother	Father	*χ* ^2^_2_	*P*_2_	Mother	Father	*χ* ^2^_3_	*P*_3_
MTHFR 677C>T	C	120 (52.6)	121 (53.1)	0.009	0.925	57 (36.5)	63 (40.4)	0.488	0.485	24 (57.1)	25 (59.5)	0.049	0.825
	T	108 (47.4)	107 (46.9)			99 (63.5)	93 (59.6)			18 (42.9)	17 (40.5)		
MTHFR 1298A>C	A	202 (88.6)	195 (85.5)	0.954	0.329	135 (86.5)	131 (84.0)	0.408	0.523	35 (83.3)	36 (85.7)	0.091	0.763
	C	26 (11.4)	33 (14.5)			21 (13.5)	25 (16.0)			7 (16.7)	6 (14.3)		
MTRR 66A>G	A	178 (78.1)	180 (78.9)	0.052	0.820	109 (69.9)	119 (76.3)	1.629	0.202	25 (59.5)	26 (61.9)	0.050	0.823
	G	50 (21.9)	48 (21.1)			47 (30.1)	37 (23.7)			17 (40.5)	16 (38.1)		

Note: *χ*^2^_1_ and *P*_1_, *χ*^2^_2_ and *P*_2_, and *χ*^2^_3_ and *P*_3_ all represent the allele distribution analysis between mother and father within the same subject group.

**Table 4:** Allele distribution differences of MTHFR 677C>T, MTHFR 1298A>C, and MTRR 66A>G polymorphisms in either mothers or fathers between control and CHD (VSD and non-VSD).

Genes	Alleles	Parent	Non-VSD (78) versus control (114)				VSD (21) versus control (114)			
			*χ* ^2^	*P*	OR	95% CI	*χ* ^2^	*P*	OR	95% CI
MTHFR 677C>T	T versus C	Mother	9.654	0.002	1.930	1.272–2.928	0.290	0.590	0.833	0.429–1.619
		Father	5.973	0.015	1.669	1.105–2.521	0.595	0.441	0.769	0.394–1.501
MTHFR 1298A>C	C versus A	Mother	0.365	0.546	1.209	0.653–2.235	0.916	0.339	1.554	0.626–3.854
		Father	0.174	0.677	1.128	0.641–1.984	0.001	0.975	0.985	0.385–2.520
MTRR66 A>G	G versus A	Mother	3.298	0.069	1.535	0.965–2.442	6.539	0.011	2.421	1.213–4.833
		Father	0.382	0.537	1.166	0.716–1.898	5.696	0.017	2.308	1.147–4.645

Note: for exact number of each allele see Table 3.

**Table 5:** Genotype distribution differences of MTHFR 677C>T, MTHFR 1298A>C, and MTRR 66A>G polymorphisms between control and CHD (VSD and non-VSD) mothers.

	MTHFR 677C>T			MTHFR 1298A>C			MTRR 66A>G		
	CC	CT	TT	AA	AC	CC	AA	AG	GG
Control	36 (31.6)	48 (42.1)	30 (26.3)	89 (78.1)	24 (21.1)	1 (0.8)	67 (58.8)	44 (38.6)	3 (2.6)
Non-VSD	12 (15.4)	33 (42.3)	33 (42.3)	57 (73.1)	21 (26.9)	0 (0)	37 (47.4)	35 (44.9)	6 (7.7)
VSD	8 (38.1)	8 (38.1)	5 (23.8)	14 (66.7)	7 (33.3)	0 (0)	7 (33.3)	11 (52.4)	3 (14.3)
Non-VSD versus control	*χ*2	3.288	8.473		0.828	0.638		1.433	3.396
	*P*	0.070	0.004		0.363	1.000		0.231	0.138
	OR	2.063	3.300		1.366	0.989		1.440	3.622
	95% CI	0.937–4.542	1.454–7.488		0.697–2.679	0.967–1.011		0.792–2.621	0.856–15.330
VSD versus control	*χ*2	0.278	0.215		1.457	0.157		2.92	8.340
	*P*	0.598	0.643		0.227	1.000		0.088	0.004
	OR	0.750	0.750		1.854			2.393	9.571
	95% CI	0.257–2.189	0.222–2.535		0.673–5.107			0.862–6.643	1.615–56.736

**Table 6:** Genotype distribution differences of MTHFR 677C>T, MTHFR 1298A>C, and MTRR 66A>G polymorphisms between control and CHD (VSD and non-VSD) fathers.

	MTHFR 677C>T			MTHFR 1298A>C			MTRR 66A>G		
	CC	CT	TT	AA	AC	CC	AA	AG	GG
Control	37 (32.5)	47 (41.2)	30 (26.3)	82 (71.9)	31 (27.2)	1 (0.9)	68 (59.6)	44 (38.6)	2 (1.8)
Non-VSD	12 (15.4)	39 (50.0)	27 (34.6)	55 (70.5)	21 (26.9)	2 (2.6)	47 (60.2)	25 (32.1)	6 (7.7)
VSD	8 (38.1)	9 (42.9)	4 (19.0)	16 (76.2)	4 (19.0)	1 (4.8)	7 (33.3)	12 (57.1)	2 (9.5)
Non-VSD versus control	*χ*2	5.778	5.931		0.001	0.855		0.389	3.553
	*P*	0.016	0.015		0.976	0.741		0.533	0.130
	OR	2.559	2.775		1.010	2.982		0.822	4.340
	95% CI	1.176–5.566	1.206–6.385		0.527–1.936	0.264–33.689		0.444–1.522	0.839–22.441
VSD versus control	*χ*2	0.052	0.544		0.484	1.575		3.782	6.221
	*P*	0.820	0.461		0.487	0.313		0.078	0.013
	OR	0.886	0.617		0.661	5.125		2.649	9.714
	95% CI	0.311–2.519	0.169–2.247		0.205–2.133	0.305–86.248		0.968–7.247	1.179–80.024
